# Bean and chia development in accordance with fertilization management

**DOI:** 10.1016/j.heliyon.2021.e07316

**Published:** 2021-06-12

**Authors:** Jaqueline Calzavara Bordin-Rodrigues, Tiago Roque Benetoli da Silva, Debora Fernandes Del Moura Soares, Juliana Stracieri, Rhaizza Lana Pereira Ducheski, Gessica Daiane da Silva

**Affiliations:** a Universidade Estadual de Maringá (UEM), Campus Umuarama, Paraná, Brazil; b Programa de Pós-Graduação em Ciências Agrárias. Estrada da Paca, São Cristóvão, 87501-970, Umuarama, Paraná, Brazil

**Keywords:** Phaseolus vulgaris, Residual fertilization, Salvia hispanica, Off-season

## Abstract

Chia seed is expanding on the market due to its characteristics, but there are few studies on its response to residual fertilization of other crops. The objective was to evaluate the vegetative and productive parameters of common bean as a function of the base fertilization increment and to verify the influence of the residue of this fertilization on the development of chia. The experiment was carried out in two stages, Maringá State University, Umuarama Regional Campus, in a randomized block design with 4 replications. The treatments for the first stage were: T1 - doses recommended for beans and T2, T3, T4 and T5, were recommended doses for beans with increments for each treatment. The evaluated variables were: shoot dry matter, number of pods per plant, grains per plant, grains per pod, 1000 grains weight and yield. In the second stage, the experiment was installed in the same place of the previous cultivation. The treatments were: residual bean fertilization, T6 - plus the treatment with the recommendation for chia. The evaluated variables were: macro and micronutrient leaf contents, shoot dry matter, final plant population, 1.000 grains weight, oil content and yield. For beans and chia, soil samples were collected after harvest to evaluate chemical attributes. In common bean, the results were not significant in the evaluated parameters. In soil, the residual effect of beans was significant for P and K, with 27.2 mg dm^−3^ and 167.70 mg dm^−3^, in treatment T5 and chia was 23.1 mg dm^−3^ and 89.7 mg dm^−3^, for treatment T6, respectively. In chia, yield, oil content and P for leaf macro and micronutrient leaf contents were significant. Thus, the vegetative and productive parameters of the common bean were not influenced by the increase in fertilization. The residual effect was higher for P and K, for beans and chia. For chia, influences by residual effect were observed.

## 1 Introduction

Beans (Phaseolus vulgaris L.) are considered one of the most important legumes for human consumption, with about 23 million hectares of producing areas in the world (Souza, 2013). According to (2019) Brazil, in the 2017/2018 harvest, produced about 1,280.9 thousand tons in the first crop, and about 1.21 million tons in the second crop, an increase of 1.2% in relation to the 2016/2017 harvest.

To achieve good yield, beans need to be fertilized according to the technical recommendations for cultivation, which improves the nutritional values of the plant (Pereira et al., 2014). The requirement becomes important due to the export of nutrients exerted by the plant.

In fertilization, not all the amount of nutrients applied is absorbed by the plant, part remains in the soil, being called residual fertilization (Souza et al., 2010; Dickmann et al., 2017). Galvao et al. (2013) observed an increase in bean grain yield, using the residual effect of fertilization on sorghum cultivation. Souza et al. (2010), when evaluating this form of utilization, observed the positive effect of residual fertilization used in bean cultivation on Brachiaria brizantha. The effects vary according to the requirements of the species that will succeed the bean (Dickmann et al., 2017).

An alternative to succeeding in bean cultivation may be chia. This culture is gaining space in the market for its nutritional characteristics, economic and productive potential. The chia moves about $ 50 billion per year in one hectare, can yield 200 to 2500 pounds (Busilacchi et al., 2013; Migliavacca et al., 2014), and be marketed the seed of between 10 to 15 real (Everitt, 2012). In Argentina, in 2008, chia production contributed approximately 4% of world grain production, with chia responsible for 24% of the agricultural industry in this country (Lema, 2010).

Chia seeds contain protein (15–25%), fat (30–33%), carbohydrate (26–41%), dietary fiber (18–30%), minerals, vitamins and dry matter (90–93%). in its composition (Ixtaina et al., 2008) and can be used in food and diets, as it has omega 3 fatty acid contents and antioxidant properties (Jamboonsri et al., 2012). In addition, the seeds do not have gluten (Bueno et al., 2010), which makes chia an important alternative to be incorporated into the diet.

For chia cultivation, the fertilization employed is based on the amounts used in the field (Malavolta, 2006; Guerra et al., 2006). In Argentina, fertilization has been carried out with about 15–45 kg and 37 kg ha^−1^ of nitrogen and phosphorus respectively. In Mexico, doses of 68 kg ha^−1^ of nitrogen carried out at sowing are reported (Ayerza and Coates, 2006).

However, there is still little information on proper fertilization, cultivation techniques for this plant, and the influence of residual fertilization of other crops on its development and production. Thus, the present work aims to evaluate the productive and vegetative parameters of common bean as a function of the increase of base fertilization and to verify the influence of the residue of this fertilization on the development of chia.

## 2 Material and methods

The experiment was carried out at the Regional Campus of the State University of Maringá (UEM), in the municipality of Umuarama, Paraná, in the geographic coordinates 23°47′30.7″ south latitude and 53°15′23.3″ west longitude, at an altitude of 407 m. The region has a Cfa or subtropical climate, according to the Köppen classification (Instituto Agronomico do Parana, 2014).

The soil of the area is classified as dystrophic Red Latosol (Empresa Brasileria de Pesquisa Agropecuaria, 2018) of sandy texture. Prior to the installation of the experiment, the soil chemical analysis of the area was collected (Table 1), with the aid of an auger at two points in each experimental plot, at a depth of 0–20 cm. The samples were sent to the Laboratory of Environmental and Industrial Chemistry, State University of Western Paraná, for the analysis of the chemical attributes of the soil, as follows: pH, phosphorus, calcium (Ca), magnesium (Mg) and potassium (K).

**Table 1:** Chemical attributes of the soil before the implementation of the experiment, in the 0–20 cm layer.

pH	P	M.O.	Ca	K	Mg	Al	CTC	V
CaCl_2_	mg dm^−3^	g dm^−3^	cmol_c_ dm^−3^					%
4.9	9.7	12.99	1.57	0.12	0.70	0.45	5.37	44.54

P and K extracted with resin; Organic matter extracted by the Walkley-Black method; Ca, Mg and Al extracted with KCl 1 cmol L^−1^.

The experiment was conducted under field conditions, with a randomized complete block design with four replications and five treatments. The experimental unit consisted of four streets, two meters long, and the streets were spaced 0.45 m apart. Considered as a useful area the two central streets, with 0.5 m at both ends being neglected. The experimental unit and useful area were used for cultivation and evaluation of bean and chia variables.

The treatments consisted of the recommended bean fertilization, according to the recommendation of the Paraná State Fertilization and Limitation Manual (Pauletti and Motta, 2017) for the crop, using simple N (Urea), P_2_O_5_ (Triple Superphosphate) fertilization, K_2_O (Potassium Chloride), being 20-90-50 respectively. The bean treatments were: basic fertilization, that is, the recommended dose for common bean (T1); basic fertilization +20% of fertilization (T2); basic fertilization +40% of fertilization (T3); basic fertilization +60% fertilization (T4) and basic fertilization +80% fertilization (T5). Bean sowing was performed on October 19, 2018, with soil emergence at five days after sowing.

Cultural treatments were carried out throughout the development of common bean and chia crops to keep the area free of weeds through periodic manual weeding. Pest and disease control for beans was carried out with a single preventive application of Piraclostrobin-based chemical (Comet®), diluting 0.007 L of the product in 4 L of water and spraying it throughout the experimental area. For chia, this control was with the application of the chemical based on fipronil, performed in a single application.

Beans were harvested manually when the pods and leaves of the plant were straw-stained, that is, the crop was in the R9 stage, and was carried out on January 4^th^ 2019. The variables evaluated were: shoot dry matter (kg ha^−1^), yield components (number of pods per plant, number of grains per plant, number of grains per pod), 100 grains weight (g) and yield (kg ha^−1^).

To determine the dry matter of the bean area, all the plants present in 1 m of a street were collected in the useful area of the plot, then separated the aerial part and the root system, placed in paper bags and dried in forced ventilation oven at 55–65 °C. After 48 h the weighing was performed and the data extrapolated to kg ha^−1^.

To determine the productive components, ten plants were collected in the useful area. Pods were counted and threshed and grains counted, determining the number of grains per plant, grains per pod and pod per plant.

For yield, a 1 m street was harvested in the useful area of each experimental plot, the pods were threshed and the heavy grains were converted to kg ha^−1^. Grains were separated to determine the 100 grains weight by weighing two subsamples for each repetition. Both evaluations were standardized to 13% humidity (Brasil, 2009).

After the bean harvest, soil samples were taken from each experimental plot to analyze the chemical attributes of the soil. The samples were collected on February 7, 2019 and sent to the Environmental and Industrial Chemistry Laboratory of the University. State of Western Paraná, for the analysis of chemical attributes.

To evaluate the utilization of residual bean fertilization by chia, the plots were installed in the same place. Sowing was carried out on April 1, 2019, manually, using about twenty-five seeds per meter, 3 kg ha^−1^.

The treatments used were the same used for bean cultivation (T1; T2; T3; T4; T5), but with an additional treatment, the residual fertilization plus the recommended fertilization for chia (T6), consisting of 20, 60 and 60 kg ha^−1^ of N, P_2_O_5_ and K_2_O at sowing, respectively. Considering that there is no recommendation for fertilizer for chia, we used as a basis the recommendations for mint through the Manual of Fertilization and Liming of the state of Paraná (Pauletti and Motta, 2017), since it belongs to the same botanical family.

At 64 days after planting, three plants were collected per plot. With the collection, the shoot was separated from the root system, placed in paper bags and taken to dry in the forced-air greenhouse, remaining for 48 h at room temperature 55 and 65 °C.

After drying and weighing, the samples were ground in the grinder of vegetable material and placed in paper bags and sent to the Laboratory of Environmental and Industrial Chemistry, State University of Western Parana, for the determination of macro and micronutrients of the aerial part.

Harvesting was done manually at the end of the crop cycle, and the aerial part of the plants was cut in the useful area of each plot, on July 17, 2019. After harvesting, the trees were threshed. grain and cleaning through 9 and 20 mesh sieves, with 2.00 mm one^−1^ and 850 μm aperture respectively, removing the impurities from the harvest. The variables evaluated were: macro (N, P, K, Ca and Mg) and aerial micronutrients (Cu, Zn, Fe and Mn) content, aerial part dry matter, final plant population, 1000 grains weight, oil content and productivity.

The dry matter of chia aerial part was determined with the same procedures used for the bean, that is, the vegetable waste generated by threshing was taken to the greenhouse, weighed and converted to kg ha^−1^. For the determination of final plant population, the harvested plants were counted in the useful area of each plot and the values converted to plants per hectare.

For the determination of the 1.000 grains weight, two samples of 200 seeds per plot and weighed in precision balance were counted, with the data collected, a static analysis was carried out, also performed with the other variables. Yield was obtained by weighing the harvested grains in the useful area and then converting them to kg ha^−1^. Both the 1.000 grains weight and the productivity had their values standardized to 13% humidity (Brasil, 2009). The oil content of the seeds was quantified in the laboratory by the hexane extraction methodology in a Soxhlet apparatus (Instituto Adilfo Lutz, 2008).

After the chia harvest, soil samples were taken from each experimental plot to analyze the chemical attributes of the soil. The samples were collected on August 7, 2019 and sent to the Environmental and Industrial Chemistry Laboratory of the University State of Western Paraná, for the analysis of chemical attributes.

Statistical analysis was performed using the variance analysis model, with 5% significance, using the Sisvar program (Ferreira, 2011). Means were compared by Tukey test at the same significance level.

## 3 Results and discussion

In beans, no difference (p ≤ 0.05) was observed for the parameters of number of grains per plant, grains per pod, pod per plant, plant population (Table 2), and shoot dry matter, of 100 grains weight and bean yield (Table 3). These results corroborate those found by Soratto et al. (2004) and Meira et al. (2005), who did not observe the influence of increasing doses (0–210 and 240 kg ha^−1^, respectively) of nitrogen on the number of grains per pod, grains per plant, 100 grains weight, yield and number of pods per plant.

**Table 2:** Number of grains per plant, grains per pod and pod per plant and final plant population, depending on the recommended fertilization. Umuarama (PR), 2019.

Treatments	Grains per plant	Grains per pod	Pod per plant	Final plant population
	number			
T1	20.6 a	3.5 a	6.1 a	230,833 a
T2	20.0 a	3.5 a	5.5 a	255,000 a
T3	20.2 a	3.7 a	5.2 a	226,677 a
T4	21.0 a	3.7 a	5.7 a	201,011 a
T5	25.5 a	4.0 a	6.7 a	219,999 a
Test F	n.s.	n.s.	n.s.	n.s.
CV(%)	13.1	9.2	19.8	11.6

T1 = Recommended dose for beans, T2 = Recommended dose for common bean with 20%, T3 = Recommended dose for common bean with 40%, T4 = Recommended dose for common bean with 60%, T5 = Recommended dose for common bean with 80%; n.s = not significant at 5% probability of error; Means followed by the same letter in the column, do not differ by Tukey's test at 5% probability of error; C.V. = coefficient of variation.

**Table 3:** Dry matter of the aerial part (kg ha^−1^), weight of 100 grains (g) and productivity of bean plants (kg ha^−1^), according to the recommended fertilization. Umuarama (PR), 2019.

Treatments	Dryness of the aerial part	Weight of 100 grains	Productivity
	kg ha^−1^	g	kg ha^−1^
T1	3,752 a	23.3 a	1,409 a
T2	3,649 a	25.2 a	1,270 a
T3	3,611 a	23.3 a	1,356 a
T4	3,558 a	24.3 a	1,401 a
T5	3,530 a	22.5 a	1,460 a
Test F	n.s.	n.s.	n.s.
CV(%)	12.8	7.7	17.7

T1 = Recommended dose for beans, T2 = Recommended dose for common bean with 20%, T3 = Recommended dose for common bean with 40%, T4 = Recommended dose for common bean with 60%, T5 = Recommended dose for common bean with 80%; n.s = not significant at 5% probability of error; Means followed by the same letter in the column, do not differ by Tukey's test at 5% probability of error; C.V. = coefficient of variation.

The results found in the present study were similar to those of Valderrama et al. (2009) and Zucareli et al. (2011) who did not observe an increase in the number of pods and of 100 grains weight, in increasing doses ranging from 0 to 120 and 150 kg ha^−1^ of P_2_O_5_, respectively. This effect can be explained by the requirement that the species has for phosphorus, which for beans is low about 0,20 kg ha^−1^ of P day^−1^, when compared to nitrogen, about 2,5 kg ha^−1^ of P N day^−1^ (Rosolem and Marubayashi 1994), besides the possibility of this nutrient not being available in the soil (Empresa Brasileria de Pesquisa Agropecuaria, 2018). Moreover, even high doses when compared to the recommended fertilization, did not become toxic to beans, allowing the development and production, without making the soil unfavorable.

In studies with increasing doses of potassium (K) the authors Sguario Júnior et al. (2006) and Rodrigues et al. (2013) found no effect of doses ranging from 0 to 120 kg ha^−1^ K_2_O on the number of pods per plant and grains per pod, grains per plant, 100 grain weight and bean yield. Possibly these results were found, because the potassium presents losses by leaching, of all applied fertilization 50–70% is leached (Wu and Liu, 2008), and when considered the soil of the place of cultivation, ie sandy, the losses can be empowered.

The results were not significant (p ≤ 0,05) for shoot dry matter, because the different fertilization dosages did not provide higher matter production. These results corroborate the authors Sguario Júnior et al. (2006), who working with different application forms and doses (0–120 kg ha^−1^) of potassium, as well as Junior et al. (2015) who worked with doses ranging from 0 to 100 kg ha^−1^ of N and P_2_O_5_.

Possibly, the fertilization employed as a function of soil analysis for common bean cultivation was sufficient to supply the plant needs (Galeazzi, 2017) not justifying the increment, which may have been fixed or adsorbed on the soil. soil colloids. Even if the increment provided higher results in the evaluated parameters, it would not justify the increase of fertilization, being explained by the Law of decreasing increments, described by Mitscherlich (Raij, 1991; Troeh et al., 2007), because it is the highest cost in cultivation, when compared to other inputs.

The average yield found in the experiment (Table 3) was 1.379 kg ha^−1^, higher than national averages in the first, second and third crop of 2018/2019, with 1,078, 930 and 1,201 kg ha^−1^, respectively, and Paraná, which were 1,527, 1,730 and 970 kg ha^−1^, in the first, second and third harvests respectively (Companhia Nacional de Abastecimento, 2019).

For the residual effect of the base fertilization (Table 4) of beans, the pH in calcium chloride (CaCl_2_), Ca and Mg, presented no significant results, since the treatments employed in the experiment did not contain Ca, Mg sources. The increase of pH values and Ca and Mg contents in the soil occurs through the application of limestone, which promotes the reduction of soil acidity and increases the levels of these nutrients (Prezotti and Guarçoni, 2013).

**Table 4:** Chemical attributes of the soil after the bean harvest, as a function of residual fertilization. Umuarama (PR), 2019.

Treatments	pH	Ca	Mg	K	P
	CaCl_2_	mg dm^−3^			
T1	5.16 a	725.4 a	479.7 a	109.2 b	12.6 d
T2	5.10 a	702.0 a	468.0 a	140.4 ab	17.8 c
T3	5.31 a	756.6 a	542.1 a	148.2 ab	22.7 b
T4	5.64 a	920.4 a	553.8 a	163.8 a	24.6 ab
T5	5.40 a	791.7 a	573.3 a	167.7 a	27.2 a
Test F	n.s.	n.s.	n.s.	∗	∗
CV(%)	7.9	18.6	10.5	17.7	26.6

T1 = Recommended dose for beans, T2 = Recommended dose for common bean with 20%, T3 = Recommended dose for common bean with 40%, T4 = Recommended dose for common bean with 60%, T5 = Recommended dose for common bean with 80%; n.s and ∗ = not significant and significant at 5% probability of error; Means followed by the same letter in the column, do not differ by Tukey's test at 5% probability of error; C.V. = coefficient of variation.

The T5 treatment presented higher amount of P, followed by the T3 and T2 treatments, being compared with the AB treatment. For potassium (K), all treatments presented higher residual effect when compared to treatment T1. Nutrient availability is related to the balance between the amount of nutrient in the soil solution (Psolution and exchangeable K) and the stored (P-labile and non-exchangeable K), but some factors such as: clay, organic matter and soil water content (Silveira and Moreira, 1990), but in sandy soils these factors are in smaller quantity, which causes the fixation and imbalance between the soil solution and the stored nutrient.

The presence of larger amounts of residual effect for P and K in T5 treatment was possibly due to the higher amount of these nutrients applied to the soil, since most of the added fertilizers in excess become immobile due to colloid adsorption (Hinsinger, 2001), thus increasing the amount in the soil, favoring succession cultivation (Reis Júnior and Fontes, 1996). In dose studies, the highest residual concentrations were observed in the plots where there was the highest fertilization (Silva et al., 2001).

For chia, there was no difference in macronutrient, nitrogen (N), potassium (K), calcium (Ca) and magnesium (Mg) levels in leaves and shoot dry matter (Table 5) between treatments. Only phosphorus was significant, and T6, and T2, T3, T4 and T5 showed significant difference when compared to the non-incremental AB treatment in the aerial part of chia. Due to the fact that P is fundamental for bean yield because it stimulates growth (Nkaa et al., 2014), however, it is little exported, remaining in the still soil, however, with the succession of chia cultivation, this nutrient was absorbed and exported.

**Table 5:** Dry matter of the aerial part (MSPA), leaf contents of nitrogen (N), phosphorus (P), potassium (K), calcium (Ca) and magnesium (Mg), depending on the fertilization management of Salvia hispanica L Umuarama (PR), 2019.

Treatments	MSPA	N	P	K	Ca	Mg
	g	g kg^−1^				
T1	6.9 a	33.8 a	2.53 b	19.1 a	11.1 a	2.9 a
T2	6.8 a	29.7 a	2.82 a	18.5 a	9.3 a	3.0 a
T3	6.5 a	29.5 a	3.39 a	18.8 a	11.3 a	3.0 a
T4	6.7 a	29.9 a	2.46 a	19.3 a	11.9 a	2.9 a
T5	7.0 a	32.8 a	2.63 a	18.3 a	9.9 a	2.7 a
T6	7.1 a	37.0 a	6.67 a	18.6 a	10.9 a	2.9 a
Teste F	n.s.	n.s.	∗	n.s.	n.s.	n.s.
CV (%)	10.7	26.1	18.6	3.1	24.3	10.9

T1 = Recommended dose for beans, T2 = Recommended dose for common bean with 20%, T3 = Recommended dose for common bean with 40%, T4 = Recommended dose for common bean with 60%, T5 = Recommended dose for common bean with 80%, T6 = Recommended dose for beans + Recommended dose for chia; n.s and ∗ = not significant and significant at 5% probability of error, respectively; Means followed by the same letter in the column, do not differ by Tukey's test at 5% probability of error; C.V. = coefficient of variation.

When nutrient requirements for chia are observed, phosphorus is one of the most required, with recommendation ranging from 15 to 37 kg ha^−1^ (Ayerza and Coates, 2006; Maciel et al., 2019; Miranda, 2012). This nutrient is part of the constitution of various plant cell compounds (Taiz et al., 2016). When observed the amount of this nutrient in chia seed, it can be found about 0.604 mg dm^−3^ seed, being the second in greater quantity, preceded only by potassium (0.826 mg md^−3^ chia seed) (Chicco et al., 2008).

For the copper (Cu), zinc (Zn), iron (Fe) and manganese (Mn) micronutrients, the results found, as well as for the macronutrients, were not observed significant influence of the treatments used (Table 6).

**Table 6:** Leaf contents of copper (Cu), zinc (Zn), iron (Fe) and manganese (Mn), and depending on the fertilization management. Umuarama (PR), 2019.

Treatments	Cu	Zn	Fe	Mn
	mg kg ^−1^			
T1	10.5 a	53.7 a	353 a	112.0 a
T2	11.2 a	64.7 a	664 a	92.5 a
T3	11.2 a	76.1 a	511 a	96.0 a
T4	12.0 a	57.2 a	528 a	72.7 a
T5	9.7 a	67.5 a	518 a	79.2 a
T6	10.0 a	68.7 a	485 a	120.0 a
Test F	n.s.	n.s.	n.s.	n.s.
CV (%)	18.5	29.7	34.2	28.9

T1 = Recommended dose for beans, T2 = Recommended dose for common bean with 20%, T3 = Recommended dose for common bean with 40%, T4 = Recommended dose for common bean with 60%, T5 = Recommended dose for common bean with 80%, T6 = Recommended dose for beans + Recommended dose for chia; n.s = not significant at 5% probability of error; Means followed by the same letter in the column, do not differ by Tukey's test at 5% probability of error; C.V. = coefficient of variation.

For the variables, final plant population, number of panicles per plant and weight of 1000 grains (Table 7), the results were not significant, possibly the doses used in the treatments were lower or higher than the chia nutritional requirement. The nutrients provided by the residual effect of bean base fertilization and the increase of fertilization in sowing, directly and indirectly participation of plant morphological structures, chlorophyll molecule, photosynthetic processes (Basi et al., 2011; Gazola et al., 2014).

**Table 7:** Final plant population, number of panicles per plant, weight of 1,000 grains (g), productivity (kg ha^−1^) and oil content in the chia grains, depending on the fertilization management. Umuarama (PR), 2019.

Treatments	Population	Plants por plant	Weight of 1.000 grains	Productivity	Oil content
	Number		g	kg ha^−1^	%
T1	455,555 a	13.0 a	1.28 a	924 b	13.8 b
T2	485,000 a	17.0 a	1.32 a	1,186 ab	14.1 b
T3	458,555 a	15.3 a	1.26 a	1,258 ab	14.8 b
T4	525,250 a	10.3 a	1.34 a	1,292 ab	14.5 b
T5	500,050 a	11.0 a	1.26 a	1,380 a	16.9 a
T6	533,333 a	18.0 a	1.27 a	1,432 a	17.9 a
Teste F	n.s.	n.s.	n.s.	∗	∗
CV (%)	34.2	42.5	7.1	24.3	7.3

T1 = Recommended dose for beans, T2 = Recommended dose for common bean with 20%, T3 = Recommended dose for common bean with 40%, T4 = Recommended dose for common bean with 60%, T5 = Recommended dose for common bean with 80%, T6 = Recommended dose for beans + Recommended dose for chia; n.s and ∗ = not significant and significant at 5% probability of error, respectively. Means followed by the same letter in the column, do not differ by Tukey's test at 5% probability of error. C.V. = coefficient of variation.

With the limitation of nutrients, it generates interference in the size of all morphological parts of the plant (Taiz et al., 2016), ie in smaller amounts of panicles and grain weight and in higher doses the nutrients are directed to the development vegetative (Chan, 2016).

This culture response to mineral fertilization varies according to many factors, as well as genotype, cultivar, fertilizer type, application time, dose, source, loss and mobility of the nutrient and organic matter available in the soil (Carrubba, 2009). The soil in the area is classified as sandy, which has low organic matter rates and nutrient losses from leaching or specific fixation are higher when compared to other soil types.

In field studies, with increasing doses from 0 to 120; 80 and 120 kg ha^−1^ N, P and K, respectively, were not observed influences, for the number of panicles, in Tocantins, with application rates N (Chan, 2016), plant population, with application of P (Novais and Silva, 2016) and weight of 1000 grains, with K application (Silva et al., 2018), in Paraná, corroborating the results found. The doses used in the experiments of these authors and the present study did not increase seedling emergence or made the area unfavorable for chia vegetative and reproductive development (Novais and Silva, 2016).

High coefficients of variation were also observed for panicle number and final chia population; however, discarding variables with such values is an unnecessary procedure (Storck et al., 2010), since the number of repetitions, plot size, experimental design, environmental heterogeneity and genetic diversity can all influence (Chan, 2016).

Many of these factors could be minimized by the development of varieties adapted to the growing environment through breeding programs (Cahill, 2004), as chia is grown in various climates.

There was a significant influence of treatments on grain yield (Table 7), with the highest averages found in T6 and T5 treatments, with 1,432 and 1,380 kg ha^−1^ respectively, when compared with T1, with 924 kg ha^−1^. There was also significant response in the oil content, especially the treatments T6 and T5, which presented 17.9 and 16.9%, respectively.

The significantly higher values observed in these treatments are possibly related to the amount of fertilizer applied to the crop and the residual effect of the nutrients in the soil, despite the low retention capacity of the sandy soil (Fidalski et al., 2013), because when observed the treatments with the largest increments, they also presented the highest values of productivity and oil content.

In the residual effect of the high doses, with T5, part of the nutrients may have remained immobile in the soil and made available to chia at the time of cultivation, possibly at the time of greatest demand of the species (Ayerza and Coates, 2009; Ayerza, 2010), because there was an increase in yield and oil content, thus making the results of T5 with residual effect statistically equal to those of T6 with fertilization performed at the time of sowing. This shows that even in soils with low nutrient retention capacity, higher doses promote longer retention of ions.

Similar to that obtained by some producers (1,200 kg ha^−1^), with fertilizer use, and lower than the average found in studies, with the use of fertilizer and irrigation, about 2,500 kg ha^−1^, Argentina (Coates, 2011), even under different cultivation conditions.

It is noteworthy that, in previous studies, in the same place, the average yield found was 1,115 kg ha^−1^ (Silva et al., 2018), demonstrating that, even with little difference between the means of the studies, it is possible to observe the influence of increased fertilization in chia grain production.

Cultivation of chia in succession becomes a viable option (Migliavacca et al., 2014), as chia responded both to the supplementation of residual fertilization (T6) and the use of residual fertilization (T5). It is noteworthy that the financial viability of this application was not evaluated in this work, requiring further study on this issue.

With the evaluation of soil samples collected after chia cultivation, it was possible to verify that among the chemical parameters evaluated (Table 8), only P and K were significant, with the greatest residual effect generated by T6 treatment with 23.1 mg dm^−3^ of P and 89.7 mg dm^−3^ of K, when compared to the other treatments, since these nutrients, before chia cultivation, already existed in the soil, as residual effect of bean fertilization. Thus, with the fertilization in the sowing of chia, the residual effect was complemented with this fertilization.

**Table 8:** Soil chemical attributes after harvesting of chia, depending on the residual fertilizer. Umuarama (PR), 2019

Treatamentos	pH	Ca	Mg	K	P
	CaCl_2_	mg dm^−3^			
T1	5,.35 a	655.2 a	444.6 a	058.5 b	12.2 c
T2	5.26 a	773.2 a	471.9 a	58.5 b	13.7 c
T3	5.52 a	639.6 a	448.5 a	62.4 b	14.2 c
T4	5.48 a	670.8 a	436.8 a	62.4 b	13.9 c
T5	5.46 a	643.5 a	413.4 a	70.2 b	19.6 b
T6	5.32 a	717.6 a	429.0 a	89.7 a	23.1 a
Test F	n.s.	n.s.	n.s.	∗	∗
CV(%)	5.9	20.1	16.3	22.5	14.2

T1 = Recommended dose for beans, T2 = Recommended dose for common bean with 20%, T3 = Recommended dose for common bean with 40%, T4 = Recommended dose for common bean with 60%, T5 = Recommended dose for common bean with 80%, T6 = Recommended dose for beans + Recommended dose for chia; n.s. and ∗ = not significant and significant at 5% probability of error. Means followed by the same letter in the column, do not differ by Tukey's test at 5% probability of error. C.V. = coefficient of variation.

For P, these results were found, possibly due to the amount applied and the high P adsorption capacity in the soil colloids (Caione et al., 2013). For K, even with low exchangeable capacity (Allison et al., 2001) and ease of loss by leaching, part remained in the soil as a residual effect (Brady and Weil, 2013). With the amount of fertilizer employed before and after chia cultivation, it is possible to observe that the availability of nutrients continued as a residual effect and may increase over time (Sousa et al., 2004), because with the application of higher doses, higher amounts of P and K are available for the next crops to be implanted (Parente et al., 2016).

Silva et al. (2018), when evaluating the residual effect of mineral fertilization at the doses of 300, 600, 900 and 1,200 kg ha^−1^, observed that this effect remained until the end of the experiment, due to the amount of fertilization applied at the beginning and the permanence of the fertilizers nutrients in the soil at the end of the experiment.

For CaCl_2_, Ca and Mg (Table 8), the results were similar to those of Table 4, ie, not significant. The pH levels, Ca and Mg, are high when lime is applied, because with the use of this soil concealer, there is the release of OH^−^ that react with H^+^ and Al^+3^ present in the soil, increasing the pH value, in addition to making Ca and Mg available by neutralizing soil acidity (Rodrigues et al., 2017).

## 4 Conclusion

For the cultivation of common bean, no influence of the increment of the basic fertilization in the productive and vegetative parameters was observed.

The residual effect of common bean crop was expressed in higher amount of nutrients P and K in the soil, in treatment T5. In chia cultivation, the nutrients P and K in the treatment T6.

For chia cultivation, the residual effect influenced the P of the macro and micronutrients of the shoot and for yield and oil content in the treatments T6 and T5.

## Declarations

### Author contribution statement

All authors listed have significantly contributed to the development and the writing of this article.

### Funding statement

The authors thank the Coordenação de Aperfeiçoamento de Pessoal de Nível Superior - Brasil (CAPES) for the scholarship. This study was financed in part by the 10.13039/501100002322Coordenação de Aperfeiçoamento de Pessoal de Nível Superior - Brasil (CAPES) - Finance Code 001.

### Data availability statement

Data included in article/supplementary material/referenced in article.

### Declaration of interests statement

The authors declare no conflict of interest.

### Additional information

No additional information is available for this paper.
